# Hypoglossal and lumbar motor neuron death in old Sprague–Dawley rats

**DOI:** 10.14814/phy2.71061

**Published:** 2026-08-18

**Authors:** Sang Won Cheung, Laney Bjerke, Debanjali Dasgupta, Gary C. Sieck, Matthew J. Fogarty

**Affiliations:** ^1^ Department of Physiology and Biomedical Engineering Mayo Clinic Rochester Minnesota USA; ^2^ Department of Neurology Mayo Clinic Rochester Minnesota USA

**Keywords:** aging, hypoglossal, lumbar, motor neurons, stereology

## Abstract

Striated muscle weakness and motor neuron (MN) death are associated with aging. These alterations affect both brainstem and spinal motor pools, including hypoglossal MNs innervating the tongue and lumbar MNs innervating the lower/hindlimbs. Both MNs and striated muscles exhibit type‐selective motor unit vulnerability, where larger MNs (likely fast fatigable motor units) and type IIx/b muscles are vulnerable to death and atrophy, respectively. These findings have been cemented in humans to some degree and in Fischer 344 and Wistar rats. However, MN survival in old age has not been evaluated in Sprague–Dawley rats. Here, in 6‐ (young) and 24‐month‐old (old) female and male Sprague–Dawley rats, we performed stereological MN counts on hypoglossal and lumbar MNs using 16 μm Nissl‐stained sections. We observed a 35% loss of hypoglossal and lumbar MNs in old age. Although mean MN somal surface areas were moderately reduced in hypoglossal and unchanged in lumbar MNs with age, frequency histogram analyses revealed a disproportionate loss of larger MN in old age hypoglossal and lumbar pools. These findings reveal reliable and robust observations of MN death in rats. This neurogenic aspect of neuromotor impairment with age may be a key driver of sarcopenia and frailty in the elderly.

## Graphical abstract

Hypoglossal and lumbar motor neurons (MNs) surival is essential for maintiaining neuromotor health into old age. We observed loss of predominantly larger hypoglossal and lumbar MNs in 24 compared to 6 months old Sprague‐Dawley rats. This shows MN loss in old age is common between rat strains and is similar to elderly humans.

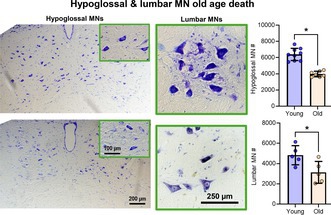

## 1 INTRODUCTION

Hypoglossal and lumbar motor neurons (MNs) innervate muscles of the tongue and the hind/lower limbs, respectively. In old age, the selective atrophy and reduced muscle force generation (sarcopenia) (Larsson et al., 2019; Sieck & Fogarty, 2025) closely resemble the denervation effect (Geiger et al., 2001; Miyata et al., 1995; Rowan et al., 2012). In humans, tongue muscle weakness may predispose the elderly to dysphagia and aspiration pneumonia, serious contributors to morbidity and mortality (Crow & Ship, 1996; Ebihara et al., 2012; Janssens & Krause, 2004; Martin et al., 1994; Nativ‐Zeltzer et al., 2022). Likewise, age‐related limb muscle weakness may impair the capacity for movement, diminish quality of life and increase risk of fall, a major contributor to morbidity and mortality (Landi et al., 2012; Larsson et al., 1979; Larsson et al., 2019). There is increasing evidence and recognition that sarcopenia and other age‐associated neuromotor dysfunctions may be due to death of MNs and denervation of target muscle fibers (Cheung & Fogarty, 2026; Doherty et al., 1993; Drey et al., 2014; Hepple & Rice, 2016; Larsson et al., 2019; Willadt et al., 2018). In humans, generalized lumbar MN loss in old age is well documented (Cruz‐Sanchez et al., 1998; Kawamura et al., 1977; Terao et al., 1996; Tomlinson & Irving, 1977), while in the rat, both hypoglossal (Fogarty, 2023; Fogarty et al., 2026) and lumbar (Jacob, 1998; Rooker et al., 2026; Rowan et al., 2012) MN death is evident. Strikingly, despite numerous MN assessments being performed in Wistar, and inbred Fischer 344 (F344) and F344‐Brown Norway hybrids (Cheung & Fogarty, 2026), only one study assessing cervical MN numbers in aged outbred Sprague–Dawley rats has been published (Das et al., 2016).

In humans and rats, neither death of MNs nor the effects of sarcopenia appear uniform across all motor unit types (Fogarty, Mantilla, & Sieck, 2019; Larsson et al., 2019; Sieck & Fogarty, 2025). Instead, much like the major age‐associated neurodegenerative condition affecting MNs, Amyotrophic Lateral Sclerosis (ALS) (Dukkipati et al., 2018; Fogarty, 2018a; Kiernan & Hudson, 1991; Nijssen et al., 2017), Fast Fatigable (type FF) motor units are afflicted, while Slow (type S) and Fast Fatigue‐Resistant (type FR) units are resilient (Larsson et al., 1979; Sieck & Fogarty, 2025). In ALS patients and preclinical species, MN pathophysiological changes are predominantly in larger (likely type FF) MNs (Dukkipati et al., 2018; Fogarty, Mu, et al., 2020a; Kaplan et al., 2014; Kiernan & Hudson, 1991; Leroy et al., 2014; Pun et al., 2006). This is mirrored in aging rats, where pathophysiological alterations are mostly confined to larger MNs (Cheung & Fogarty, 2026; Christensen & Fogarty, 2026; Fogarty, 2023; Fogarty et al., 2018; Fogarty et al., 2026; Fogarty & Sieck, 2023; Jacob, 1998; Kawamura et al., 1977).

It remains unknown if hypoglossal and lumbar MN survival differs in aging outbred Sprague–Dawley rats, similar to observations in inbred rat strains (Cheung & Fogarty, 2026). Here we aimed to determine the survival and size of hypoglossal and lumbar MNs in female and male 6‐ (young) and 24‐month‐old (old) Sprague–Dawley rats. We hypothesize that hypoglossal and lumbar MN survival will be reduced in old age and that this reduction will disproportionately affect larger MNs.

## 2 MATERIALS AND METHODS

### 2.1 Ethical approval

All procedures were performed in accordance with the American Physiological Society's Animal Care Guidelines, the National Institutes of Health (NIH) guide for the use and care of laboratory animals (NIH Publications #8023, revised 1978) and were approved by the Institutional Animal Care and Use Committee (IACUC) at Mayo Clinic.

### 2.2 Experimental animals

In the present study, 16 Sprague–Dawley wild type rats (8 females and 8 males) were obtained from our breeding colony at weaning and aged to either 6 (*n* = 9; 5F/4M) or 24‐month‐old (*n* = 7; 3F/4M). Animals were maintained two animals per cage under an alternating 12:12 h light–dark cycle with ad libitum access to rat chow (Rodent Laboratory Diet Cat # 5001, Lab Diet) and fresh water.

### 2.3 Tissue collection, processing and MN counting

Rats were deeply anesthetized with intraperitoneal injection of ketamine (80 mg/kg) and xylazine (20 mg/kg), then euthanized by exsanguination and perfused with heparinized saline followed by perfusion with 4% paraformaldehyde (PFA) in 0.1 M phosphate‐buffered saline (PBS, pH 7.4). The fixed brainstem and lumbar spinal cord were then excised and post‐fixed in 4% PFA in PBS overnight, then immersed overnight in 25% sucrose in PBS. Serial transverse 16 μm cryosections of the brainstem and lumbar spinal cord were cut and stained with 0.1% thionin (v/v) in an acetic acid buffer (Fogarty, 2023; Fogarty et al., 2013; Fogarty et al., 2015).

Hypoglossal and lumbar MN numbers were quantified bilaterally in the hypoglossal nucleus and unilaterally (right side) in the lumbar motor enlargement, with landmarks identified by rat brainstem and spinal cord atlases (Paxinos et al., 1999; Watson et al., 2009). Hypoglossal MN counts were performed stereologically on every 5th section and lumbar MN counts on every 10th section in a manner identical to previous studies (Fogarty, 2023; Fogarty et al., 2013; Fogarty et al., 2015) on a Zeiss Axoskop II equipped with a motorized stage 20× air objective (1.0 NA, Zeiss, Gottingen, Germany), with the entire hypoglossal nucleus (Fogarty, 2023) or lateral motor column examined as a single counting field per section (Rooker et al., 2026). As previously described, MNs were counted only if they were large cells (the mean of the long and short axis >15 μm) (Dobbins & Feldman, 1995; Ferrucci et al., 2018; Fogarty, 2023; Odutola, 1976), with a dark cytoplasm, a distinct pale nucleus and dark nucleoli (Ferrucci et al., 2018; Fogarty, 2023; Lance‐Jones, 1982). Fragments of neurons, without a nucleus and nuclei are not counted to avoid “double counting”, consistent with stereological principles (Ferrucci et al., 2018; Slomianka, 2020).

To estimate the hypoglossal and lumbar MN somal surface areas, the *x* and *y* radii were measured using Image J (Schneider et al., 2012), with surface areas calculated assuming a prolate spheroid shape (Fogarty, 2023; Fogarty et al., 2018; Fogarty, Dasgupta, et al., 2023; Fogarty & Sieck, 2023; Ulfhake & Cullheim, 1988).

### 2.4 Statistical analysis

All statistical analyses were performed using PRISM software (GraphPad PRISM Version 10.2.3, La Jolla, CA). Study numbers were determined by sample size power analysis (*α* = 0.05, *β* = 0.95), with studies in F344 rats serving as pilot data for mean and standard deviation for young hypoglossal (4050 ± 422) and lumbar (4900 ± 426) MNs, with an expected biologically relevant difference of 20%, yielding a group size of *n* = 7 for hypoglossal and *n* = 5 for lumbar. Statistical significance was established at the *p* < 0.05 level, reported precisely to three decimal places. All data are presented as mean ± the standard deviation (SD) and assessed for normality with Shapiro–Wilk tests. For all age comparisons, Student's unpaired *t*‐test, Mann–Whitney *U*‐Tests, or 2‐Way ANOVAs were used. To ensure statistical differences were meaningful, reliable, and robust, we reported Cohen's *d* (Gandevia, 2021). Experiments, imaging, and analyses were performed blind to age and sex. We did not power to detect sex differences in aging and included female and male rats to mirror the heterogeneity of the aging human population. Recent studies have shown a lack of sex dimorphism in many MN properties, including size (Christensen & Fogarty, 2026; Fogarty, 2023; Fogarty, 2025; Fogarty, Rana, et al., 2023).

## 3 RESULTS

### 3.1 Rat characteristics

When stratified by sex, we observed a significant effect of sex (*F*_(1,14)_ = 5.0; *p* = 0.042) and age (*F*_(1,14)_ = 7.2; *p* = 0.018) on body mass (Two‐Way ANOVA) in Sprague–Dawley rats, with males (young: 636 ± 12 g; old: 799 ± 97 g) 30%–50% heavier than females (young: 345 ± 11 g; old: 511 ± 10 g) within each age group (young: *p* < 0.001; old: *p* < 0.001; Bonferroni post‐tests). Old females were ~45% heavier than young females (*p* = 0.007; Bonferroni post‐test) and old males were ~25% heavier than young males (*p* = 0.008; Bonferroni post‐test).

### 3.2 Age‐related loss of hypoglossal MNs

**FIGURE 1:**
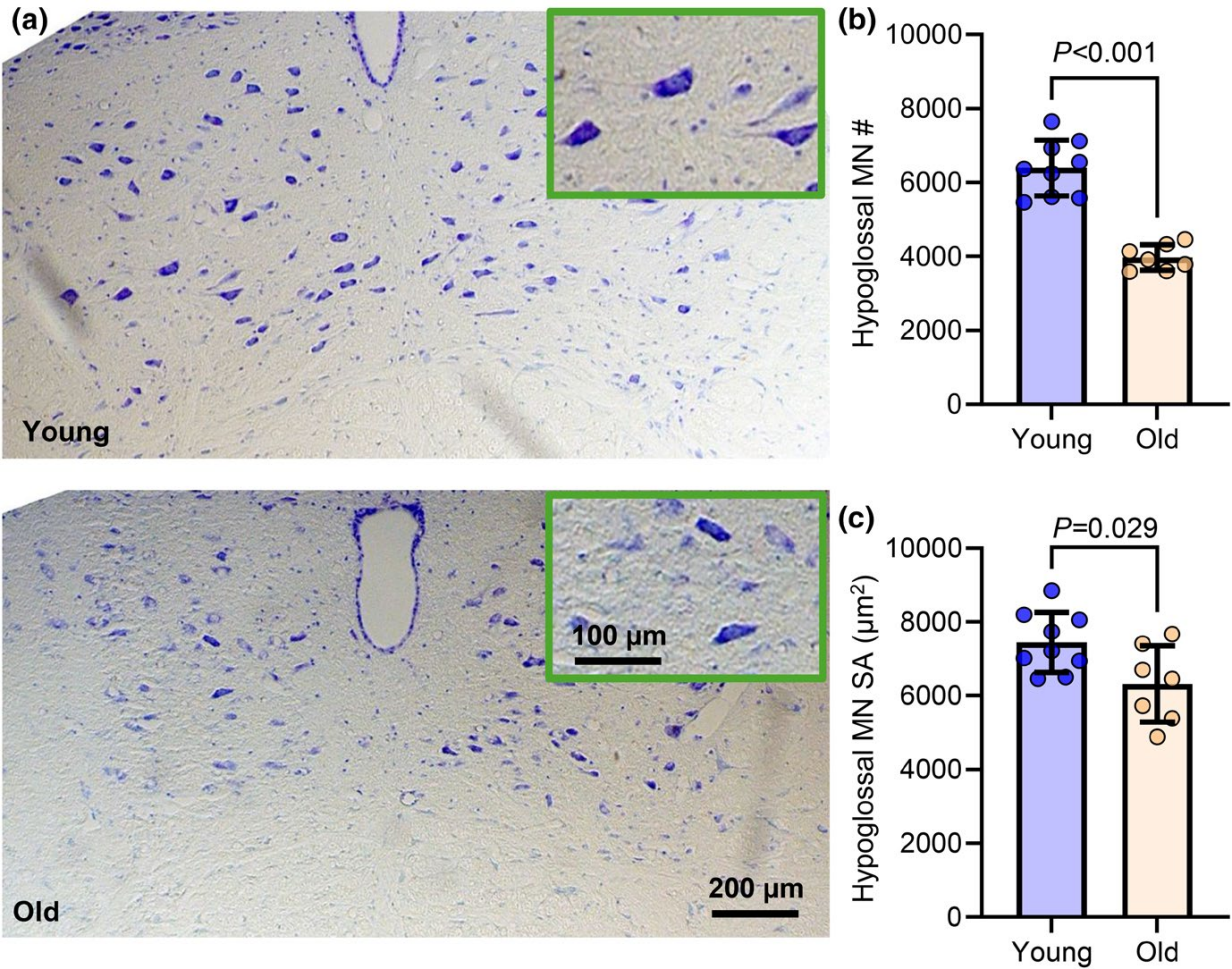
(a) Representitive pictomicrographs of transverse sections through the brainstem showing the hypoglossal nucleus of a young and an old Sprague–Dawley rat. Insets show larger magnification hypoglossal MNs. (b) Scatterplot showing reduced hypoglossal MNs in old compared to young rats. (c) Scatterplot showing reduced hypoglossal MNs somal surface area in old compared to young rats. All analyses Student's unpaired *t*‐tests, each dot represents an individual rat (the “*n*”), where *n* = 9 young and *n* = 7 old.

Hypoglossal MN numbers were evaluated in the entirety of the bilateral hypoglossal nuclei (Figure 1a). In older rats, the number of hypoglossal MNs was reduced by ~38% compared to young Sprague–Dawley rats (3974 ± 345 vs. 6392 ± 757; *p* < 0.001, Student's unpaired *t*‐test; Cohen's *d* = 3.93; Figure 1b). When stratified by sex, the effect of age on MN survival remained (*F*_(1,12)_ = 52.5; *p* < 0.001), but no effect of sex was observed (*F*_(1,12)_ = 0.3; *p* = 0.602; 2‐Way ANOVA).

### 3.3 Larger hypoglossal MNs are more vulnerable to aging

Hypoglossal MN somal surface area was evaluated for the entire bilateral hypoglossal MN pool (Figure 1). In older rats, mean hypoglossal MN surface areas were reduced by ~15% compared to young F344 rats (6321 ± 1037 μm^2^ vs. 7443 ± 815 μm^2^; *p* = 0.029, Student's unpaired *t*‐test; Cohen's *d* = 1.20; Figure 1c). When stratified by sex, the effect of age on MN surface area remained (*F*_(1,12)_ = 6.4; *p* = 0.027), but no effect of sex was observed (*F*_(1,12)_ = 0.3; *p* = 0.593; 2‐Way ANOVA).

**FIGURE 2:**
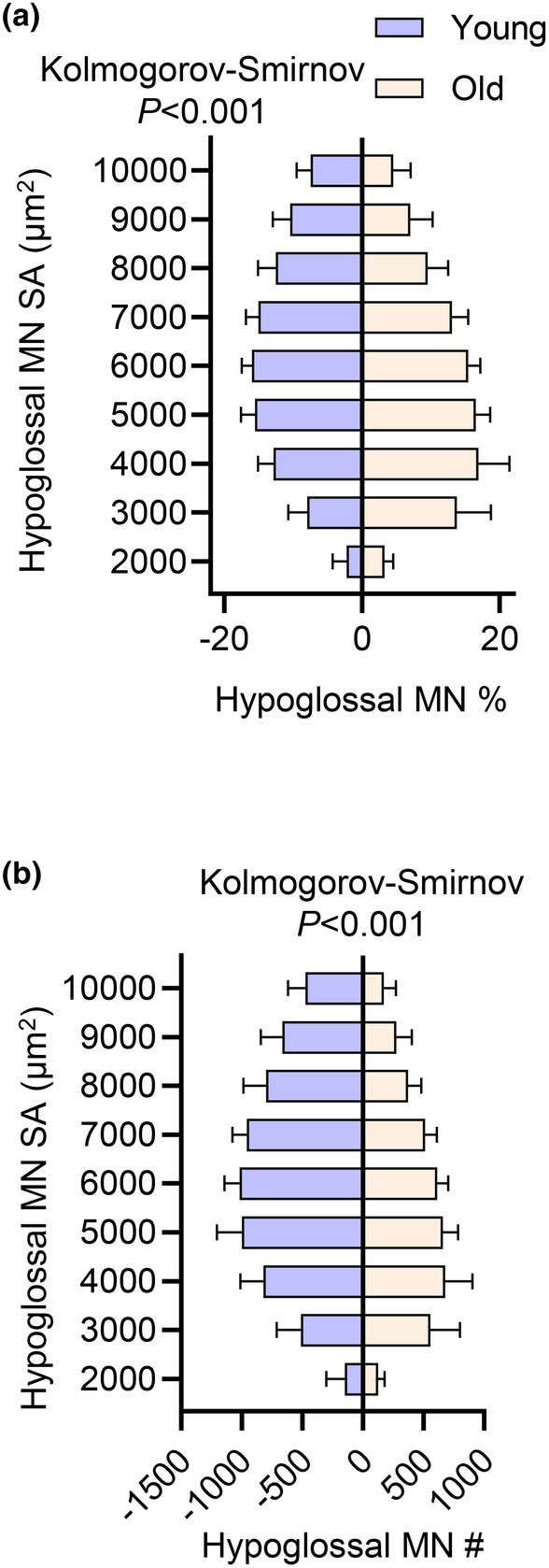
(a) Frequency histogram shows reduced % larger hypoglossal MNs size distributions (somal surface area) in old compared to young Sprague–Dawley rats. Kolmogorov–Smirnov test, *p* < 0.001. (b) Frequency histogram shows reduced number of larger hypoglossal MNs (somal surface area) in old compared to young Sprague–Dawley rats. Kolmogorov–Smirnov test, *p* < 0.001.

Frequency histograms showed an altered distribution of hypoglossal MN somal size with age‐associated reductions in the proportion of larger MNs (Kolmogorov–Smirnov test, *p* < 0.001; Figure 2a). Frequency histograms showed an altered distribution of hypoglossal MN somal size with age‐associated reductions in the relative number of larger surviving MNs (Kolmogorov–Smirnov test, *p* < 0.001; Figure 2b).

### 3.4 Age‐related loss of lumbar MNs

**FIGURE 3:**
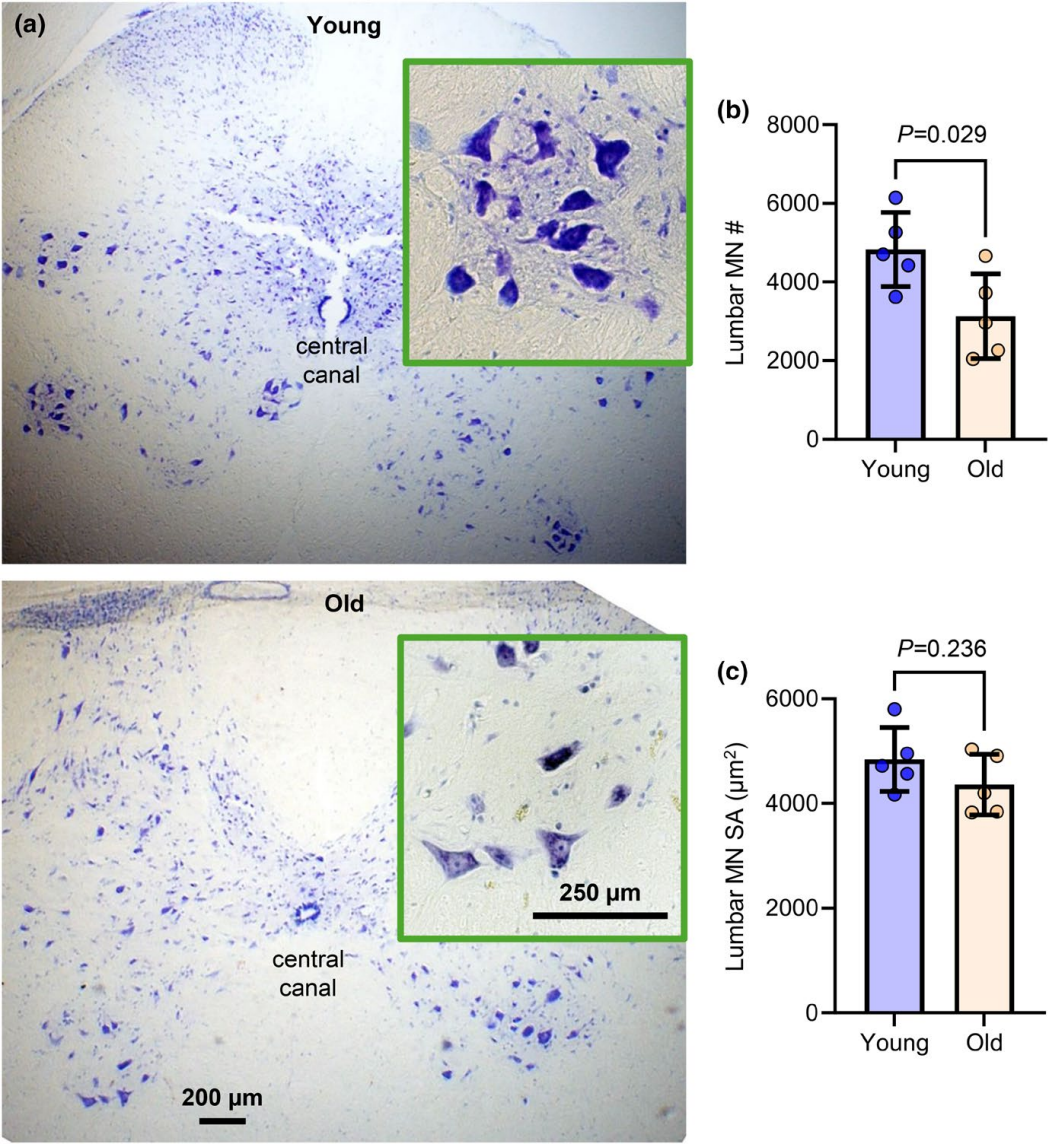
(a) Representitive pictomicrographs of transverse sections through the lumbar spinal cord showing the MNs within the lateral motor column of a young and an old Sprague–Dawley rat. (b) Scatterplot showing reduced lumbar MNs in old compared to young rats. (c) Scatterplot showing unchanged lumbar MN somal surface area in old compared to young rats (*p* = 0.236). All analyses Student's unpaired *t*‐tests, each dot represents an individual rat (the “*n*”), where *n* = 5 young and *n* = 5 old.

Lumbar MN numbers were evaluated in the entirety of the left and right lumbar lateral motor columns in young (*n* = 5) and old (*n* = 5) Sprague–Dawley rats (Figure 3a). In older rats, the number of lumbar MNs was reduced by ~35% compared to young Sprague–Dawley rats (3128 ± 1080 vs. 4828 ± 942; *p* = 0.029, Student's unpaired *t*‐test; Cohen's *d* = 1.68; Figure 3b).

### 3.5 Larger lumbar MNs are more vulnerable to aging

Gross lumbar MN somal surface area was unchanged between young (4841 ± 607 μm^2^) and old (4361 ± 577 μm^2^) Sprague–Dawley rats (*p* = 0.236, Student's unpaired *t*‐test; Figure 3c).

**FIGURE 4:**
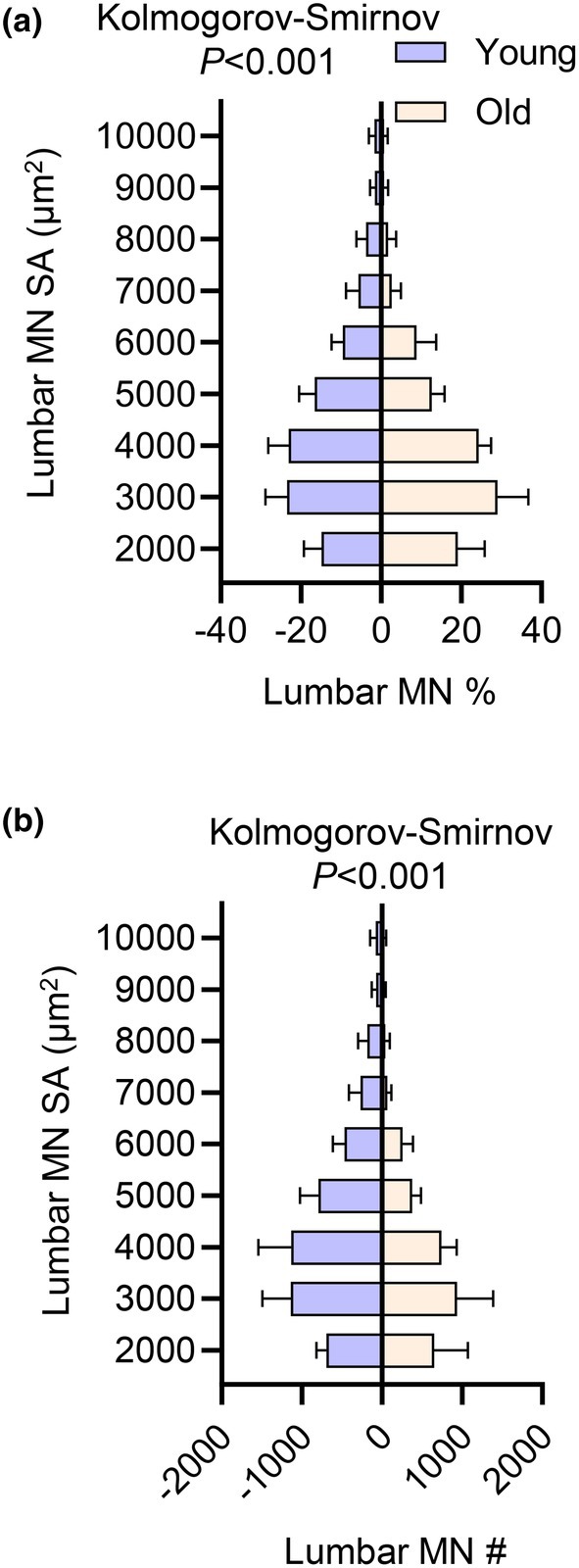
(a) Frequency histogram shows reduced % larger lumbar MNs size distributions (somal surface area) in old compared to young Sprague–Dawley rats. Kolmogorov–Smirnov test, *p* < 0.001. (b) Frequency histogram shows reduced number of larger lumbar MNs (somal surface area) in old compared to young Sprague–Dawley rats. Kolmogorov–Smirnov test, *p* < 0.001.

When lumbar MN somal surface areas were plotted as a frequency histogram within each animal, a reduction in the frequency of larger lumbar MNs was readily apparent (*p* < 0.001, Kolmogorov–Smirnov test; Figure 4a). Frequency histograms showed an altered distribution of lumbar MN somal size with age‐associated reductions in the relative number of larger surviving MNs (Kolmogorov–Smirnov test, *p* < 0.001; Figure 4b).

## 4 DISCUSSION

In the present study, we found an overall loss of hypoglossal and lumbar MNs, with MN loss disproportionately affecting MNs with larger somal surface areas. These results are consistent with pathological findings in human spinal cord and in the brainstem and spinal cord of rats. Age‐related MN loss is also consistent with denervation underlying atrophy and force reduction observed in old skeletal muscle, which predominantly affects type IIx/b fibers (Fogarty, Gonzalez Porras, et al., 2019; Hepple & Rice, 2016; Kemp et al., 2026; Larsson et al., 2019), although this was not determined in the present Sprague–Dawley cohort. Motor unit type‐specific denervation in aging is congruent with behavioral deficits in maneuvers that require recruitment of brainstem or spinal cord type FF motor units and conservation of those that require only S and FR units (Doherty et al., 1993; Fogarty, 2025; Fogarty et al., 2026; Fogarty & Sieck, 2026; Khurram et al., 2018; Larsson et al., 1979; Larsson et al., 2019; Rowan et al., 2012; Sieck & Fogarty, 2025). However, only the latter resilience of S and FR units has been borne out behaviorally in old Sprague Dawley rats (Fogarty & Sieck, 2026). Overall, these findings provide a robust trans‐strain platform underscoring the important neurogenic contribution MNs make to impaired motor function in old age.

We observed a marked loss of hypoglossal and lumbar MNs in old Sprague–Dawley rats, with death of ~35% of hypoglossal and lumbar MNs. These observations are consistent with generalized Sprague–Dawley cervical MN loss (Das et al., 2016) and loss of MNs within brainstem motor nuclei and the spinal cord in old age humans and rats (Cheung & Fogarty, 2026). Studies showing generalized MN loss are complemented by those showing selective loss of individual muscle motor pools (Fogarty et al., 2018; Fogarty & Sieck, 2023; Hashizume et al., 1988; Hashizume & Kanda, 1990; Hashizume & Kanda, 1995; Ishihara et al., 1987; Kanda, 2002; Pannerec et al., 2016). In the present study, size distributions of hypoglossal and lumbar MNs indicated a disproportionate loss of larger MNs. In Sprague–Dawley rats, the age‐related reduction in the number of larger hypoglossal MNs is consistent with evaluations of MNs in other rat strains (Fogarty et al., 2018; Fogarty & Sieck, 2023; Hashizume et al., 1988; Kanda & Hashizume, 1998). In the hypoglossal nucleus, where we had sufficient numbers, hypoglossal MN survival and size were not affected by sex. Overall mean size differences in lumbar MNs may be obscured if the orientation of the material prepared for MN counting is not optimal for MN size assessment, which requires parasagittal sections (Sterling & Kuypers, 1976). Further, the orientation may explain why hypoglossal MN somal SA estimates are larger than lumbar MNs in the same rats, as the maximal extent of hypoglossal MNs is in the transverse orientation assessed here (Altschuler et al., 1991; Kanjhan et al., 2016), while lumbar MN maximal size is not reflected in transverse sections used here (Sterling & Kuypers, 1976).

Currently, there are a few molecular markers of slow and fast MN types, with most used in mouse (Manuel & Zytnicki, 2019). For type S MNs, estrogen‐related receptor beta (ESRRB) (Enjin et al., 2010), synaptic vesicle protein 2A (SV2A) (Chakkalakal et al., 2010), calcium‐activated potassium channel (SK3) (Deardorff et al., 2013; Dukkipati et al., 2018) and ubiquitin carboxy‐terminal hydrolase L1 (UCHL1) (Yasvoina et al., 2013) have been suggested as a potential markers of S MNs (Manuel & Zytnicki, 2019). Delta‐like 1 homolog (DLK1) (Muller et al., 2014), calcitonin gene‐related peptide ((CGRP)/calca) (Piehl et al., 1993), Chondrolectin (Chodl) (Enjin et al., 2010), and Matrix metallopeptidase 9 (MMP‐9) (Dukkipati et al., 2018; Kaplan et al., 2014) have been considered markers of type FF MNs. However, none of the molecular markers have been unequivocally shown to strictly adhere to the Size Principle, where motor units are recruited in an orderly size‐dependent manner affecting input resistance and capacitance (Dukkipati et al., 2018; Fogarty, 2018b; Leroy et al., 2014; Manuel & Zytnicki, 2019). This is important as the main determinant of neuronal surface area (and consequently input resistance and capacitance) are dendrites (Aitken & Bridger, 1961; Kanjhan et al., 2016; Rall, 1959; Rall et al., 1992), which have been shown to be somal size dependant in hypoglossal and lumbar MNs (Christensen & Fogarty, 2026; Fogarty, Mu, et al., 2020a; Fogarty, Mu, et al., 2020b). Whether these and other intrinsic properties of MNs scale with molecular slow and fast marker expression is largely lacking, particularly in rats and cats, where the size heterogeneity is larger than in mice (Manuel et al., 2019), clearly evidenced in retrogradely labeled populations of individual spinal motor pools (e.g., phrenic MNs (Brandenburg et al., 2020; Fogarty et al., 2018)).

In the major age‐associated disease of MNs, ALS, a size‐dependent loss and degeneration of larger brainstem and spinal cord MNs is observed in patients (Kiernan & Hudson, 1991) and rodent models (Dukkipati et al., 2018; Fogarty et al., 2024; Hegedus et al., 2008). Prior to MN death, synaptic and mitochondrial dysfunctions are prevalent in ALS (Cozzolino & Carri, 2012; Fogarty, 2018a; Fogarty, 2019). In aging, similar pathophysiological findings seem to be apparent, with synaptic and mitochondrial dysfunctions occurring in MNs prior to their demise (Fogarty, 2025; Fogarty et al., 2026). These synaptic deficits may be related to coordination deficits in old age, rather than weakness per se (Fogarty, 2026; Ingram et al., 2023; Lord et al., 2016). In ALS, mitochondrial dysfunctions are a potent driver of pathophysiology (Bendotti et al., 2001; Martin et al., 2007; Sasaki et al., 2004; Sasaki & Iwata, 2007; Wong et al., 1995). In aging, we have shown that mitochondrial deficits occur in larger MNs and type IIx/b muscle fibers vulnerable to sarcopenia (Brown et al., 2021; Christensen & Fogarty, 2026; Fogarty et al., 2026; Fogarty, Marin Mathieu, et al., 2020). It should be noted that systemic inflammatory cytokines, the trigger of mitochondrial dysfunction in aging, steadily escalate across the lifespan of F344 rats (Fogarty et al., 2026). This inflammaging phenomenon seems to cause brainstem mitochondrial alterations prior to muscle changes (Fogarty et al., 2026). In addition, this generalized inflammaging is sufficient to drive MN and muscle fiber pathophysiology FF motor units (i.e., larger MNs and type IIx/b fibers) in F344 rats (Christensen & Fogarty, 2026; Fogarty et al., 2026). The relative salience of synaptic and mitochondrial influences, and their interactive effects on age‐related neuromotor decline are areas of intense study.

One of the major factors governing the use of different rat strains is their rate of continuous growth throughout their lifespan, with the current outbred Sprague–Dawley strain much larger than inbred F344 and F344 hybrid rats (Elliott et al., 2016; Turturro et al., 1999), including those used in past MN evaluations (Fogarty, 2023; Fogarty, 2025; Fogarty et al., 2018; Fogarty & Sieck, 2023). In the present study, rats increased in body mass with age, whereas in the more commonly used F344 aging, 24‐month‐old rats are either losing weight or have stable body mass. In addition, detailed survival curves have been obtained for F344 and F344 hybrids, with 24‐month‐old being the point where 50% of all F344 rats are dead (Turturro et al., 1999; Yuan et al., 2009), which approximates to 75–80 human years at current life expectancy (Weon & Je, 2012). Here we show aged outbred Sprague–Dawley rats exhibit MN death, similar to other rat strains (Cheung & Fogarty, 2026), although we do not know the equivalent human age. Another consideration is whether different muscle groups and behaviors are affected with different severities based on neurodegenerative burden. We speculate that rather than magnitude of weakness, the severity of the behavioral consequences contributes most to organisimal morbidity and mortality, with tongue muscles required for life‐sustaining feeding and breathing (along with speech). For example, hindlimb weakness in human ALS sufferers may lead to individuals being slow to locomote or wheelchair bound, whilst airway and swallow impairment result in a more rapid clinical decline and risk of death (Adamske et al., 2021; Borges et al., 2022; Garand et al., 2022; Holdom et al., 2026; Neville et al., 2026). There is also some anatomical information suggesting that subtle changes in tongue musculature due to denervation may be of use in clinical staging and response to treatment (Shaw et al., 2025). Future studies may clarify the causal or consequential relationships between tongue neuromotor pathophysiology and age‐related frailty or neurodegenerative conditions (Humbert et al., 2010; Kappert et al., 2020; Youmans et al., 2009).

In summary, our observations of hypoglossal and lumbar MN loss in Sprague–Dawley rats are entirely consistent withpast observations in other inbred and outbred rat strains (Cheung & Fogarty, 2026). This loss of MNs may lead to similar tongue and lower/hindlimbs dysfunctions previously observed in humans and other rat strains (Ansved & Larsson, 1989; Ansved & Larsson, 1990; Basken et al., 2012; Connor et al., 2009; Fogarty, 2025; Fogarty et al., 2026; Glass et al., 2021; Hassan et al., 2021; Larsson, 1995; Larsson et al., 1991; Larsson et al., 1997; Larsson et al., 2019; Larsson & Ansved, 1995; Nagai et al., 2008; Sieck & Fogarty, 2025; Suzuki et al., 2002). Hypoglossal and lumbar MN loss disproportionately affects larger (likely type FF) MNs, similar to age‐associated neurodegeneration in ALS (Dukkipati et al., 2018; Kiernan & Hudson, 1991; Nijssen et al., 2017). It is tempting to speculate similar mechanisms may be at play, particularly the interaction of major ALS candidate pathophysiological triggers such as mitochondrial dysfunction (Cozzolino & Carri, 2012), which also arise in aging (Fogarty et al., 2026; Larsson et al., 2019). This study provides a firm foundation to further evaluate mechanisms of MN loss in the context of aging and neurodegeneration in rodents.

## AUTHOR CONTRIBUTIONS

**Sang Won Cheung:** Data curation; formal analysis; investigation; visualization. **Laney Bjerke:** Investigation. **Debanjali Dasgupta:** Investigation. **Gary C. Sieck:** Data curation; funding acquisition; project administration; supervision. **Matthew J. Fogarty:** Conceptualization; data curation; formal analysis; funding acquisition; investigation; project administration; supervision; visualization.

## FUNDING INFORMATION

This work was supported by National Institutes of Health Grants AG057052, AG‐044615 (G.C.S.) and AG086136 (M.J.F.).

## CONFLICTS OF INTEREST STATEMENT

None of the authors have any conflicts of interest, real nor perceived, to disclose. The funders had no role in the preparation of this manuscript.

## ETHICS STATEMENT

All procedures were approved by Mayo Clinic IACUC (#00008010‐24) and adhered to national guidelines.

## Data Availability

Data is available from the corresponding author at reasonable request.
